# Supplementary material for: Modeling glioblastoma heterogeneity as a dynamic network of cell states
Source: Mol Syst Biol. 2021 Sep 16;17(9):e10105. doi: 10.15252/msb.202010105 (PMC8444284; doi:10.15252/msb.202010105)
Supplement: Supplementary file 6 — Source Data for Figure 5 [file MSB-17-e10105-s004.zip › Figure5A_sourcedata/GSEA_3071/nosigdb_stateI.GseaPreranked.1622795145664/RICHARDS_VERHAAK_CANCERCELL_2010_PRONEURAL.html]

Details for gene set RICHARDS\_VERHAAK\_CANCERCELL\_2010\_PRONEURAL[GSEA]

|  || Dataset | state13071 |
| Phenotype | NoPhenotypeAvailable |
| Upregulated in class | na\_neg |
| GeneSet | RICHARDS\_VERHAAK\_CANCERCELL\_2010\_PRONEURAL |
| Enrichment Score (ES) | -0.6292722 |
| Normalized Enrichment Score (NES) | -3.0876021 |
| Nominal p-value | 0.0 |
| FDR q-value | 0.0 |
| FWER p-Value | 0.0 |
Table: GSEA Results Summary

  

Fig 1: Enrichment plot: RICHARDS\_VERHAAK\_CANCERCELL\_2010\_PRONEURAL      
 Profile of the Running ES Score & Positions of GeneSet Members on the Rank Ordered List

  

| PROBE | GENE SYMBOL | GENE\_TITLE | RANK IN GENE LIST | RANK METRIC SCORE | RUNNING ES | CORE ENRICHMENT || 1 | KLRC3 |  |  | 167 | 0.879 | -0.0334 | No |
| 2 | ADGRL3 |  |  | 231 | 0.765 | -0.0385 | No |
| 3 | EPB41 |  |  | 536 | 0.488 | -0.1184 | No |
| 4 | TTC3 |  |  | 654 | 0.431 | -0.1449 | No |
| 5 | KLRK1 |  |  | 842 | 0.367 | -0.1929 | No |
| 6 | DNM3 |  |  | 870 | 0.359 | -0.1946 | No |
| 7 | MVB12B |  |  | 1238 | 0.281 | -0.2963 | No |
| 8 | MAPT |  |  | 1627 | -0.270 | -0.4044 | No |
| 9 | GADD45G |  |  | 1657 | -0.275 | -0.4081 | No |
| 10 | CDK5R1 |  |  | 1879 | -0.302 | -0.4671 | No |
| 11 | FBXO21 |  |  | 1911 | -0.307 | -0.4708 | No |
| 12 | CDC25A |  |  | 1953 | -0.313 | -0.4773 | No |
| 13 | DCAF7 |  |  | 1961 | -0.313 | -0.4740 | No |
| 14 | VEZF1 |  |  | 1970 | -0.314 | -0.4709 | No |
| 15 | CLASP2 |  |  | 2005 | -0.318 | -0.4753 | No |
| 16 | SCG3 |  |  | 2025 | -0.322 | -0.4752 | No |
| 17 | AMOTL2 |  |  | 2031 | -0.323 | -0.4711 | No |
| 18 | OLIG2 |  |  | 2044 | -0.324 | -0.4690 | No |
| 19 | GSK3B |  |  | 2048 | -0.325 | -0.4643 | No |
| 20 | CBX1 |  |  | 2096 | -0.334 | -0.4722 | No |
| 21 | ZNF711 |  |  | 2101 | -0.334 | -0.4676 | No |
| 22 | TMEFF1 |  |  | 2165 | -0.346 | -0.4799 | No |
| 23 | MARCKSL1 |  |  | 2215 | -0.356 | -0.4880 | No |
| 24 | YPEL1 |  |  | 2254 | -0.364 | -0.4928 | No |
| 25 | PAFAH1B3 |  |  | 2264 | -0.365 | -0.4891 | No |
| 26 | CRMP1 |  |  | 2324 | -0.377 | -0.4997 | No |
| 27 | ARHGEF9 |  |  | 2327 | -0.379 | -0.4938 | No |
| 28 | CSNK1E |  |  | 2353 | -0.384 | -0.4944 | No |
| 29 | ABAT |  |  | 2383 | -0.390 | -0.4961 | No |
| 30 | TOP2B |  |  | 2686 | -0.469 | -0.5758 | No |
| 31 | PHLPP1 |  |  | 2717 | -0.475 | -0.5763 | No |
| 32 | HMGB3 |  |  | 2839 | -0.516 | -0.6025 | No |
| 33 | RAD21 |  |  | 2866 | -0.527 | -0.6010 | No |
| 34 | SATB1 |  |  | 2870 | -0.527 | -0.5928 | No |
| 35 | ATAD5 |  |  | 2935 | -0.563 | -0.6017 | No |
| 36 | ZEB2 |  |  | 3031 | -0.620 | -0.6186 | Yes |
| 37 | TOPBP1 |  |  | 3033 | -0.621 | -0.6082 | Yes |
| 38 | ERBB3 |  |  | 3038 | -0.625 | -0.5985 | Yes |
| 39 | BCL7A |  |  | 3058 | -0.641 | -0.5930 | Yes |
| 40 | CDKN1B |  |  | 3065 | -0.646 | -0.5836 | Yes |
| 41 | DPF1 |  |  | 3071 | -0.651 | -0.5738 | Yes |
| 42 | STMN1 |  |  | 3171 | -0.751 | -0.5896 | Yes |
| 43 | MPPED2 |  |  | 3216 | -0.803 | -0.5886 | Yes |
| 44 | FHOD3 |  |  | 3218 | -0.806 | -0.5750 | Yes |
| 45 | PODXL2 |  |  | 3230 | -0.817 | -0.5641 | Yes |
| 46 | TMSB15A |  |  | 3241 | -0.833 | -0.5526 | Yes |
| 47 | PFN2 |  |  | 3252 | -0.852 | -0.5408 | Yes |
| 48 | MYO10 |  |  | 3255 | -0.857 | -0.5266 | Yes |
| 49 | GRIA2 |  |  | 3258 | -0.862 | -0.5123 | Yes |
| 50 | C1QL1 |  |  | 3321 | -0.967 | -0.5137 | Yes |
| 51 | GNG4 |  |  | 3323 | -0.971 | -0.4972 | Yes |
| 52 | GPM6A |  |  | 3328 | -0.976 | -0.4815 | Yes |
| 53 | TMCC1 |  |  | 3355 | -1.049 | -0.4710 | Yes |
| 54 | MCM10 |  |  | 3356 | -1.050 | -0.4529 | Yes |
| 55 | NRXN2 |  |  | 3361 | -1.072 | -0.4356 | Yes |
| 56 | DLL3 |  |  | 3368 | -1.090 | -0.4185 | Yes |
| 57 | CHD7 |  |  | 3389 | -1.167 | -0.4042 | Yes |
| 58 | SOX2 |  |  | 3394 | -1.198 | -0.3847 | Yes |
| 59 | NCAM1 |  |  | 3416 | -1.284 | -0.3687 | Yes |
| 60 | MAP2 |  |  | 3422 | -1.305 | -0.3476 | Yes |
| 61 | MMP16 |  |  | 3423 | -1.321 | -0.3248 | Yes |
| 62 | SLCO5A1 |  |  | 3441 | -1.474 | -0.3044 | Yes |
| 63 | SOX11 |  |  | 3469 | -1.784 | -0.2814 | Yes |
| 64 | ASCL1 |  |  | 3484 | -1.937 | -0.2521 | Yes |
| 65 | BEX1 |  |  | 3490 | -2.151 | -0.2164 | Yes |
| 66 | CKB |  |  | 3497 | -2.376 | -0.1772 | Yes |
| 67 | C1orf61 |  |  | 3499 | -2.534 | -0.1338 | Yes |
| 68 | DGKI |  |  | 3501 | -2.540 | -0.0903 | Yes |
| 69 | FXYD6 |  |  | 3503 | -2.596 | -0.0458 | Yes |
| 70 | DCX |  |  | 3505 | -2.772 | 0.0017 | Yes |
Table: GSEA details [plain text format]

  

Fig 2: RICHARDS\_VERHAAK\_CANCERCELL\_2010\_PRONEURAL: Random ES distribution      
 Gene set null distribution of ES for **RICHARDS\_VERHAAK\_CANCERCELL\_2010\_PRONEURAL**
